# HAPPY LIFE EXPECTANCY AND REGIONAL AND GROUP HETEROGENEITY AMONG CHINESE OLDER ADULTS

**DOI:** 10.1093/geroni/igad104.3377

**Published:** 2023-12-21

**Authors:** Chao Guo, Peisen Yang

**Affiliations:** Peking University, Beijing, Beijing, China (People’s Republic); Peking University, Beijing, Beijing, China (People’s Republic)

## Abstract

A long, healthy and happy life is the aspiration of most people and an essential part of human well-being. However, longer life expectancy does not mean a higher quality of life. The measure of quality of life includes both objective and subjective indicators. Similar to healthy life expectancy, happy life expectancy is a measure that combines life expectancy with happiness indicators and can reflect both life length and quality of life. But at present, there is relatively little research on happy life expectancy. In China, there have been no studies using panel data to calculate happy life expectancy. We obtained longitudinal data from the Chinese Longitudinal Health Longevity Survey (CLHLS). We used the multistate life table approach to calculate the happy life expectancy of Chinese older adults, and analyzed the regional and group heterogeneity. A total of 13,807 participants aged 65 and older were included in this study. We found that the proportion of happy life expectancy to remaining life of Chinese older adults increased with age, which confirmed the “compression of unhappiness” hypothesis. In addition, older adults with more education had a higher proportion of happy life expectancy than those with less education. Finally, in terms of region, older adults in the more economically developed areas of eastern China have a higher proportion of happy life expectancy. Our findings provide a more comprehensive understanding of the quality of life of Chinese older adults and highlight the importance of education for the happiness of older adults in later life.

